# Dual-Targeted Self-Assembled DNA Hydrogels Decorated With Multivalent Aptamers Loaded With DOX for Anticancer Therapy

**DOI:** 10.3389/fphar.2022.807498

**Published:** 2022-02-23

**Authors:** Fangfang Jin, Qian Zeng, Husun Qian, Dian Zhang, Yu Wei, Yange Wang, Chengsen Chai, Wei Cheng, Shijia Ding, Tingmei Chen

**Affiliations:** ^1^ Key Laboratory of Clinical Laboratory Diagnostics (Ministry of Education), College of Laboratory Medicine, Chongqing Medical University, Chongqing, China; ^2^ The Center for Clinical Molecular Medical Detection, The First Affiliated Hospital of Chongqing Medical University, Chongqing, China

**Keywords:** targeted drug delivery, DNA hydrogel, aptamer, synergistic therapy, doxorubicin

## Abstract

Chemotherapy, as one of the principal modalities for cancer therapy, is limited by its non-specific and inefficient delivery to tumors. To overcome these limitations, we report herein a dual-targeted aptamer-decorated DNA hydrogel system (DTA-H) to achieve efficient, stable, and targeted delivery of drugs. Firstly, DNA hydrogel was formed by the rolling circle amplification. By reasonable design, double target and multivalent aptamers were decorated on DNA hydrogel to load DOX. The results confirmed that DTA-H can deliver chemotherapy drugs and aptamer nucleic acids drugs to target cells, inducing degradation of HER2 protein while chemotherapy is synergistic to inhibit HER2-positive breast cancer growth. The proposed drug delivery system has significant potential to achieve efficient, stable, and targeted delivery of drugs and cancer therapy.

## Introduction

Chemotherapy [especially doxorubicin (DOX)] has been widely applied in the treatment of various cancers. Nevertheless, its non-specific and inefficient delivery to tumor tissue often leads to serious side effects and reduces the therapeutic effect (Smuder, 2019). Therefore, it is urgent to develop an ideal treatment platform with targeted and efficient drug delivery. In recent years, nanomaterials such as organic nanoparticles and polymer nanomaterials have been built into therapeutic platforms because of their strong antienzymolysis ability, easy surface functionalization, and good stability (Chen et al., 2018; Wang et al., 2021). However, due to the existence of exogenous components, these nanomaterials still face obvious challenges of cytotoxicity and immune response in the process of long-term use (Chen et al., 2018). At the same time, their complex preparation process also limits their clinical application to a great extent.

For the past few years, biocompatible deoxyribonucleic acid (DNA) nanostructures, such as DNA polyhedron, DNA origami, and DNA nanotubes, have been widely applied in biosensors, imaging, and therapy, which serve as carriers of small interference RNA (siRNA), chemotherapeutic drugs, and immunostimulatory cytosine phosphate guanine (CpG) (Jiang et al., 2019a; Jiang et al., 2019b). However, there are still some inherent defects in DNA nanostructures. DNA nanostructures are easy to degrade by endogenous nuclease *in vivo* (Keller and Linko, 2020). It is still highly desired to improve DNA biological stability. To date, DNA hydrogels have shown great potential in cancer theranostic applications, which integrates the biocompatibility, degradability, molecular identifiability, and precise programmability of DNA molecules (Jung et al., 2017; Mo et al., 2021). In particular, the serum stability of DNA hydrogel is much better than that of other DNA nanostructures (Chandrasekaran et al., 2020). Moreover, DNA hydrogel can be constructed *via* simple rolling circle amplification (RCA), an isothermal enzymatic process driven by DNA polymerase (Huang et al., 2017). The circular DNA template and short DNA primers are catalyzed by DNA ligase to synthesize long single-stranded DNA molecules with the most repetitive sequence motifs that can guide the programmable self-assembly of DNA hydrogel fabrication. Therefore, DNA hydrogel is considered as ideal drug delivery material because of its excellent biocompatibility and high biological stability.

The uptake of DNA nanoparticles by cells usually relies on passive delivery including enhanced penetration and retention effect (EPR) (Shi et al., 2020). This model may not be suitable for certain cancers that require specific targeting, such as breast cancer and lymphoma. Alternatively, molecular recognition ligands that could specifically bind to the receptor overexpressed on the surface of the target cell can accurately deliver the drug to the target cell, such as aptamers screened by systematic evolution of ligands by exponential enrichment (SELEX) (Wang et al., 2019). Besides, aptamers have many remarkable characteristics, including receptor-mediated internalization and easy integration into DNA nanostructures by the Watson–Crick concept of complementary base pairing. In particular, aptamers can be used as agonists and antagonists similar to antibodies (Nimjee et al., 2017). It has been reported that the binding of some aptamers to the target protein can induce the degradation of the target protein (Ma et al., 2019), thus improving treatment efficiency. So far, some DNA nanomaterials have been able to recognize target cells through the binding of bivalent or tervalent aptamers (Yu et al., 2018), and these DNA nanostructures have shown elevated targeting effects for target cells. However, there are still few reports on dual-targeted drug delivery mediated by multivalent aptamers.

Herein, we proposed a dual-targeted aptamer-decorated DNA hydrogel system (DTA-H) to achieve efficient, stable, and targeted delivery of drugs. The HER2 aptamer and AS1411 aptamer were easily incorporated into the DTA-H by DNA complementary base pairing. Moreover, the targeted delivery of DOX was achieved by inserting DNA base pairs as well as the G-rich quadruplex structure of AS1411, which significantly reduced the severe side effects of DOX in cancer chemotherapy (Ouyang et al., 2020). The DTA-H may hold great promise for applications in drug delivery and cancer therapy.

## Materials and Methods

### Materials

DNA sequences listed in Supplementary Table S1 were synthesized by Sangon Biotech (Shanghai, China). T4 DNA ligase and Phi29 DNA polymerase were purchased from New England Biolabs (Beijing, China). DNA marker (2000 bp) and dNTPs were purchased from Sangon Biotech. (Shanghai, China). The DMEM medium, DMEM/F12 medium, penicillin and streptomycin, and fetal bovine serum (FBS) were provided by Gibco (Grand Island, NY). CCK-8 was obtained from New Cell and Molecular Biotech (Suzhou, China).

### Cell Culture

SK-BR-3 (human HER2-positive breast cancer cell line), MCF-10A (normal human breast cell line), and MDA-MB-231 (triple-negative breast cancer cell line) were obtained from the American Type Culture Collection (ATCC; Rockville, MD, United States). SK-BR-3 cell was cultured in DMEM medium supplemented with 10% FBS and 1% penicillin–streptomycin (100 IU/ml), MCF-10A cell was cultured in MCF-10A special medium (Procell Life Science and Technology, China). MDA-MB-231 cell was cultured in DMEM/F12 medium supplemented with 10% FBS and 1% penicillin–streptomycin (100 IU/ml). All the cells were incubated at 37°C in a 5% CO_2_ humidified incubator.

### Preparation of DTA-H

DNA hydrogel was formed by RCA. The padlock (10 μM) was firstly incubated with primer (10 μM) in 1 × T4 DNA ligase buffer (50 mM Tris-HCl, 10 mM MgCl_2_, 10 mM DTT, and 1 mM ATP, pH 7.5) and 20 U/ml T4 DNA ligase at 16°C for 1 h. Then, the T4 DNA ligase was inactivated at 65°C for 10 min and the resultant mixture was mixed with 10 mM dNTPs and 10 U/ml Phi29 DNA polymerase. The RCA reaction proceeded at 30°C for 10 h. After that, the Phi29 DNA polymerase was inactivated at 65°C for 10 min and RCA product was cooled to 4°C for storage. Thereafter, the RCA products were analyzed by 1% agarose gel electrophoresis. The resultant RCA products were DNA hydrogels with certain viscoelasticity. To form aptamerdecorated DNA hydrogel, a pair of L-type aptamers (L-HER2 and L-AS1411, 10 μM) were added to the above DNA hydrogel and heated at 95°C for 5 min, then slowly annealed to room temperature. In addition, L-type aptamers with volume gradients were added to the above-mentioned DNA hydrogels with volume ratios of 1/5, 2/5, 3/5, 4/5, and 5/5 to explore the decoration of aptamers on DNA hydrogels.

### Gel Electrophoresis

Agarose gel (1%) was prepared using a 1 × TBE buffer. The gel electrophoresis was run at 120 V for 45 min in the 1× TBE buffer. To test the serum stability of the DTA-H and ssDNA, after incubating the DTA-H and ssDNA and 10% FBS supplemented growth medium at 37°C for different periods of time (0, 2, 4, 8, 16, and 24 h), the samples were subjected to gel electrophoresis as described above.

### SEM Imaging

DNA hydrogels were quick-frozen in the liquid nitrogen and then fully dried in a vacuum-freeze dryer for 9 h. The dried samples were Pt-coated with 20 mA for 10 min and characterized by using a scanning electron microscope at a voltage of 5 kV.

### Drug Loading Into DTA-H

Different volumes (0, 0.1, 0.2, 0.4, 2, 4, 10, 40, and 60 μl) of DTA-H (10 μM) were mixed with 100 μl of DOX (100 μM). The final volume was supplemented to 200 μl with sterile water. After incubation at room temperature for 24 h, the fluorescence spectra were monitored on the RF-5301PC fluorescence spectrometer (Shimadzu, Japan) by setting the excitation wavelength at 478 nm.

### Confocal Laser Imaging

The experimental procedure is as follows: approximately 1 × 10^4^ cells were plated on a 35-mm glass-bottom cell culture dish (Biosharp Life Sciences, China) and incubated with fluorescence-labeled L-HER2, L-AS1411, or DTA-H/DOX for different times at 37°C; after washed with PBS, cells were fixed with 4% formaldehyde for 20 min at room temperature. Nuclei were stained with 4′6-diamidino-2-phe-nylindole (DAPI, Boster, Wuhan, China) for 5 min using the standard protocol provided by the manufacturer. Then, the cells were washed with PBS and confocal imaging was performed with a TCS SP8 confocal laser microscope (Leica, Germany).

### Cytotoxicity Assays

The cytotoxicity of RCA product, DTA-H, free DOX, and DTA-H/DOX to different types of cells was detected by the Cell Counting Kit-8 (CCK-8) assay. According to the manufacturer’s protocol, approximately 1× 10^3^ cells (SK-BR-3 and MCF-10A cells) were plated and treated with RCA product, DTA-H, free DOX, or DTA-H/DOX for different times and concentrations at 37°C, respectively. Then, 10 μl of CCK-8 solution was added to each well and incubated for 1 h at 37°C. The absorbance at 450 nm was measured with a microplate spectrophotometer (Biotek, United States).

### Western Blotting

Cells were collected after different treatments and total protein was extracted. Firstly, cells were washed with PBS and lysed by RIPA lysis buffer supplemented with 1% protease inhibitor PMSF (Beyotime, Haimen, China). Then, protein concentration was measured by the bicinchoninic acid (BCA) protein assay reagent kit (Beyotime, Haimen, China). The electrophoresis step is to run at 70 V for 40 min and then at 100 V for 1 h. The PVDF membrane was then blocked with 5% fat-free milk powder for 2 h at room temperature. The following primary antibodies were used: HER2, phospho-AKT(Ser473), AKT, phosphor-p44/42 MAPK(Thr202/Tyr204), p44/42 MAPK, Bax, cleaved caspase-3, caspase-3 (1:1,000, Cell Signaling Technology), Bcl-2 (1:1,000, Affinity Biosciences Technology), GAPDH (1:10,000, Bioworld), and horseradish peroxidase-conjugated goat anti-mouse or rabbit immunoglobulin G (1:5,000, Beyotime, Biotechnology).

### Immunofluorescence Staining

Cells on cover-slips were washed with PBS after different treatments. After fixing with 4% formaldehyde for 20 min at room temperature, cells were incubated with primary antibodies anti-HER2 (1:200, Cell Signaling Technology) at 4°C overnight. Then, cells were washed with PBS and incubated with the anti-rabbit IgG antibodies (1:500, Alexa-594) for 1 h at room temperature. 4′6-Diamidino-2-phenylindole (DAPI, Boster, Wuhan, China) was used to stain the nuclei and fluorescence was observed with the TCS SP8 confocal laser microscope (Leica, Germany).

### Flow Cytometry

Cells in each group were treated for different times. Then, cells were digested with trypsin without ethylenediaminetetraacetic acid and collected in PBS. After being centrifugated and resuspended at a concentration of 1 × 10^6^ cells in 500 μl of PBS, cells were incubated with staining solution including DAPI (Beyotime, Haimen, China) and annexin-V APC (Sungene Biotech, Tianjin, China) in the dark. Then, cell apoptosis was detected by flow cytometry on a FACScan cytometer (BD Immunocytometry Systems, United States).

### Caspase-3 Activity Assay

Caspase-3 activity was determined using a Caspase-3 activity kit (Beyotime, Haimen, China). According to the manufacturer’s protocol, treated cells were lysed and then incubated with reaction buffer and caspase-3 substrate in 96-well microtiter plates at 37°C for 2 h; caspase-3 activity was quantified with a microplate spectrophotometer (Biotek, United States) at an absorbance of 405 nm.

### *In Vivo* Assays

All animal studies were approved by the Chongqing Management Approach of Laboratory Animal. The mouse breast xenograft tumor model was generated by subcutaneous injection of 1×10^6^ SK-BR-3 cells into the right flank of the Balb/c female nude mice aged 4–5 weeks, which were purchased from Hunan SJA Laboratory Animal Co., Ltd. (Changsha, China). When the tumor volume reached approximately 100 mm^3^, the tumor-bearing mice were randomly divided into three groups (3 mice in a group). The group treated with PBS was served as the control, while the other groups were respectively injected with L-AS1411-H/DOX (DOX: 5 mg/kg) and DTA-H/DOX (DOX: 5 mg/kg) every 4 days for 25 days. The volume of the tumor was calculated as (tumor length) × (tumor width)^2^/2, and mice body weights were measured at the same time. Mice were sacrificed at the end of the treatment. Their organs and tumor were collected for histopathology studies. Moreover, 150 μl of 100 μM L-HER2-Cy5 and DTA-H/DOX-Cy5 containing L-HER2-Cy5 was injected by tail vein injection. After 6 and 12 h, mice were anesthetized under isoflurane, and the *in vivo* fluorescence imaging was carried out by LB983 Night OWL Ⅱ from Berthold Technologies (Germany).

### Hematoxylin–Eosin and Immunohistochemistry

The tumors and main organs of tumor-bearing nude mice were collected after the mice were sacrificed, and fixed in 4% formaldehyde, embedded in paraffin, and then sliced into 4-μm sections, and then they were used for routine hematoxylin–eosin (H and E) staining. After deparaffinating and immunostaining with primary and secondary antibodies, the sections were observed with microscopy (Nikon ECLIPSE Ti-s, Japan). Additionally, the terminal deoxynucleotidyl transferase deoxyuridine triphosphate (dUTP) nick-end labeling (TUNEL) assay was used to further study the apoptosis of tumors.

## Results and Discussion

### Design of DTA-H

**SCHEME 1:**
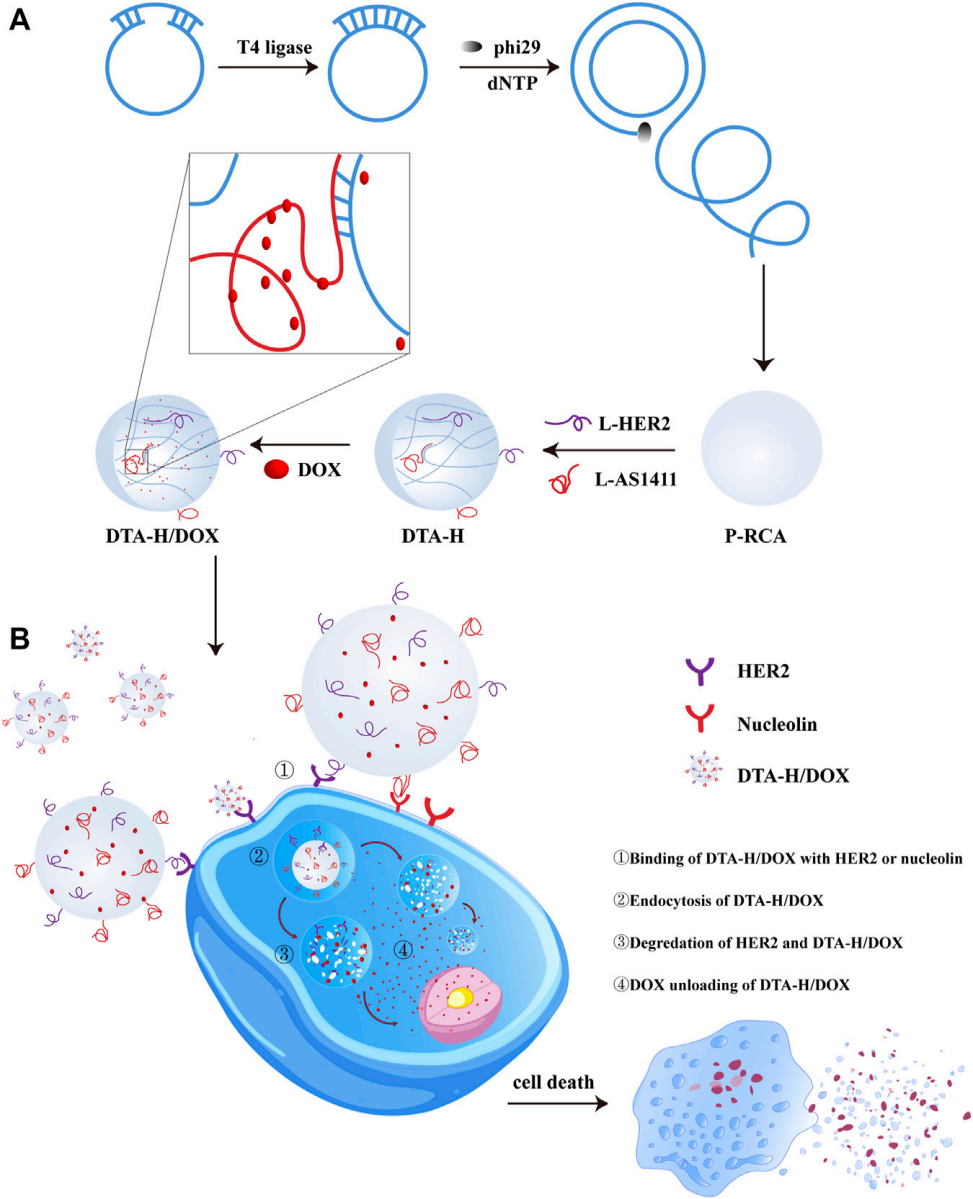
Illustration of DTA-H self-assembly and targeted cancer therapy. **(A)** RCA reaction formed DNA hydrogel, then multivalent aptamer and DOX were loaded into DNA hydrogel. **(B)** DTA-H/DOX specifically recognized and entered target cells for targeted delivery of anticancer drugs and aptamer nucleic acids to HER2-positive breast cancer cells.

In this study, DNA hydrogel was formed by RCA reaction. Here, we designed two sequences (the red part of Supplementary Table S1) in the template strand to form the long single-stranded RCA product with repetitive sequence motifs which can complement the L-HER2 aptamer and L-AS1411 aptamer (the green part of Supplementary Table S1) respectively. As shown in Scheme 1A, after DNA hydrogel annealing, L-HER2 and L-AS1411 aptamer can be decorated on DNA hydrogel to form dual-targeted DNA hydrogels decorated by multivalent aptamers (DTA-H). HER2 aptamers could specifically recognize HER2 protein on the cell membrane surface, such as SK-BR-3 cell (human HER2-positive breast cancer cell line). It is reported that HER2 breast cancer accounts for about 20%–25% of breast cancer, and the expression level of HER2 protein is 100 times higher than that of normal cells (Cooke et al., 2001). Besides, AS1411 aptamer could specifically bind to nucleolin (a protein overexpressed in many tumor types) (Trinh et al., 2015). In particular, AS1411 aptamer can load enough DOX by its G-rich G quadruplex structure (Taghdisi et al., 2018).

The above aptamer-decorated DNA hydrogels were used as the drug carriers to load DOX. In Scheme 1B, DOX-loaded DNA hydrogels (DTA-H/DOX) can specifically recognize SK-BR-3 cells and internalize into the cytoplasm, then DOX is gradually released into the cytoplasm in the lysosome and spreads to the nucleus to play an anticancer role.

#### Characterization of DTA-H and Assembly Optimization

**FIGURE 1:**
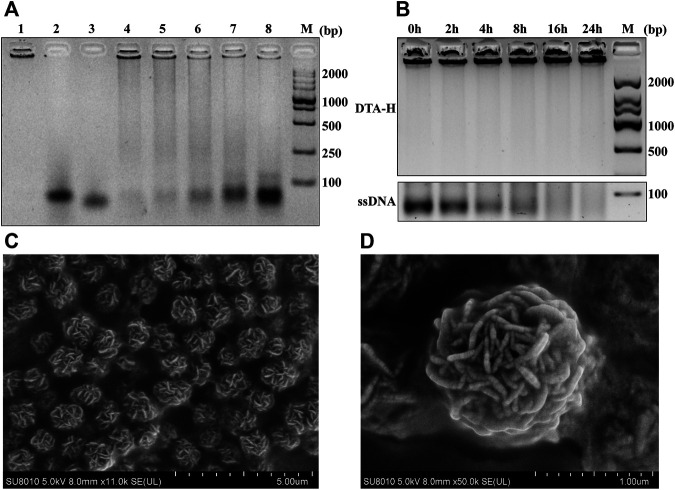
Assembly feasibility of DTA-H and SEM characterization. **(A)** Optimization of RCA product (P-RCA)/L-type HER2 aptamer (L-HER2)/L-type AS1411 aptamer (L-AS1411) ratio (P-RCA:LHER2:L-AS1411) employed during assembly of DTA-H. M represents DNA markers with 2,000-bp increments; Lanes 1–3 represent P-RCA, L-HER2, and L-AS1411; Lanes 4–8 show that the volume ratio of L-HER2:L-AS1411:P-RCA is equal to 1:1:5, 2:2:5, 3:3:5, 4:4:5, and 5:5:5, respectively. **(B)** Gel electrophoresis (1%) analysis of serum stability of DTA-H. **(C, D)** are SEM images of DTA-H hydrogel. Scale bar: 5 and 1 μm.

The assembly feasibility of DTA-H was verified by gel electrophoresis. As shown in Supplementary Figure S1A, compared with the single-strand L-HER2, the DNA hydrogel (RCA product) had almost no migration, which is consistent with other reports (Jiang et al., 2019a; Ye et al., 2020). Then, we continued to verify the decoration of L-type aptamers on RCA products (P-RCA). As shown in Supplementary Figure S1B, when the volume ratio of L-HER2:P-RCA = 3:5, L-HER2 was successfully assembled into DNA hydrogel. Then, Figure 1A showed the optimization of the volume ratio of two L-type aptamers and RCA products. It can be seen that with the increase of two L-type aptamers, the aptamer band gradually appeared on the gel. When the volume ratio of L-HER2:L-AS1411:P-RCA was less than or equal to 3:3:5, no obvious aptamer band was shown, suggesting that aptamers were completely connected to the DNA hydrogel. However, when the ratio of L-HER2:L-AS1411:P-RCA was more than 3:3:5, the aptamer band appeared, indicating an excess of aptamers did not bind to DNA hydrogel. We proved once again that the L-type aptamer can be wholly decorated on the DNA hydrogel when the ratio of L-HER2:L-AS1411:P-RCA is equal to 3:3:5 in Supplementary Figure S1C. Therefore, the ratio of L-HER2:L-AS1411:P-RCA less than or equal to 3:3:5 is the appropriate proportion of aptamer-decorated DNA hydrogel.

To better verify the formation of hydrogel, the morphology of hydrogel was observed by the naked eye. Supplementary Figure S2 showed that the DNA hydrogel formed after the reaction, making the solution turbid compared to the solution before the reaction. Also, scanning electron microscopy (SEM) was applied to observe the external and internal structures of P-RCA and DTA-H hydrogel (Supplementary Figure S3 and Figure 1). Figure 1C showed the spherical structure of the dried hydrogel sample (external structure), while the internal structure of the sphere was DNA nanoflowers (Figure 1D). These structures indicate the successful synthesis of DNA nanohydrogels.

#### Serum Stability Analysis of DTA-H

Remarkable biological stability is an essential feature of drug carriers. Chemical modifications were previously applied to prevent the nuclease digestion of DNA nanostructure, which can easily lead to biotoxicity. Thus, it is vital to evaluate the biostability of DTA-H because no chemical modification is employed in the DTA-H. Figure 1B shows that the band of ssDNA disappeared within less than 4 h, while the DTA-H can exist for almost 24 h, revealing that the DNA hydrogel improves the structural stability of DNA.

### Targeted Drug Delivery by DTA-H and Selective Cytotoxicity of DTA-H/DOX

#### DOX Loading

DOX can preferentially intercalate into dual-stranded 5′-GC-3′ or 5′-CG-3′ sequences, resulting in the quenching of its fluorescence (Yu et al., 2019). Therefore, DOX was chosen to be loaded into DTA-H. In Supplementary Figure S4, as the equivalent of DTA-H increased, the fluorescence of DOX was gradually quenched. When the molar ratio of DTA-H/DOX was lower than 50/1, the fluorescence of DOX was significantly quenched with almost no change, which verifies the high drug loading capacity of DTA-H. The ratio of DTA-H/DOX was 50/1 for subsequent studies.

#### Targeted Anticancer Drug Delivery

In order to obtain the targeting recognition ability of target cells, we designed a dual-targeting strategy to target HER2-positive breast cancer cells. The HER2 aptamer decorated on the DNA hydrogel can target the cell membrane protein HER2, while the AS1411 aptamer can target the nucleolin. Therefore, HER2 aptamer and AS1411 aptamer were selected as the dual targeting part to improve targeting efficiency, and SK-BR-3 cells were selected as target cells.

**FIGURE 2:**
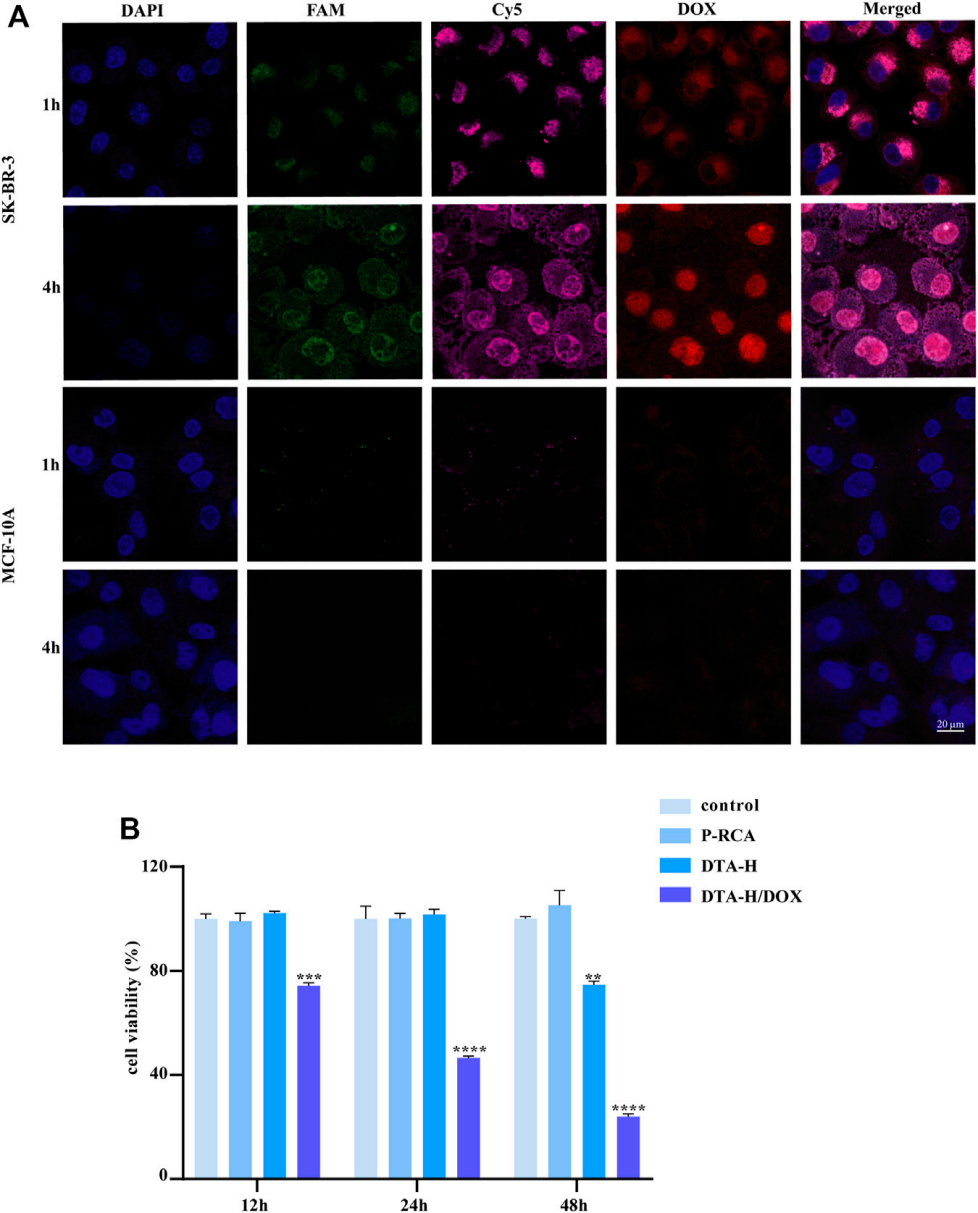
Targeted drug delivery by DTA-H and selective cytotoxicity of DTA-H/DOX. **(A)** Confocal laser images of SK-BR-3 and MCF-10A cells. Scale bar: 20 μm. **(B)** Cytotoxicity profiles of P-RCA, DTA-H, and DTA-H/DOX. Error bars denote standard deviations from three independent experiments. Statistical analysis: **p < 0.01 vs. control; ***p < 0.001 vs. control; ****p < 0.0001 vs. control.

The intracellular behaviors of DTA-H/DOX were observed by CLSM imaging, where MCF-10A (normal breast cells) were set as control cells. The optimal incubation time had been observed for 4 h (Supplementary Figure S5). Meanwhile, the dual targeting of DTA-H to target cells SK-BR-3 was proved. Supplementary Figure S6 showed the co-localization of L-HER2 and L-AS1411 in SK-BR-3 cells (both HER2 and nucleolin positive) and MDA-MB-231 cells (only nucleolin positive) in the DTA-H group, while the fluorescence was stronger in SK-BR-3 cells than in MDA-MB-231 cells, which was suspected to be due to the existence of dual targeting in SK-BR-3 cells. Also, from Figure 2A, strong fluorescence of L-HER2 and L-AS1411 decorated on DTA-H on the cell membrane was monitored and was co-located in SK-BR-3 cells rather than in MCF-10A cells. Meanwhile, the red fluorescence of DOX is restored after being released from the internalized DTA-H in the intracellular environment. DOX was initially co-localized with DTA-H and finally unloaded and distributed in other intracellular areas in SK-BR-3 cells. These results suggested that the DTA-H/DOX was first bound to the membrane proteins (HER2 or nucleolin), internalized into the cell *via* receptor-mediated endocytosis, and then mainly distributed in the cytoplasm and nucleus. It has been reported that the unloading of DOX from internalized DNA nanocarriers was through simple diffusion and affected by intracellular factors such as pH, ionic environment, and nuclease degradation (Zhu et al., 2013). Thus, DOX could rapidly release from the DTA-H under the acidic environment of endosomes and then diffuse into the cytoplasm and the nucleus to play an anti-cancer effect.

#### Selective Cytotoxicity

The enhanced binding affinity for specific cells and the high drug loading capacity of DTA-H make it a potential therapeutics nano-vehicle. The cytotoxicity of vehicle DNA hydrogel to target cells was studied firstly; Supplementary Figure S7A shows that DNA hydrogel alone had no inhibitory effect on cell proliferation within 48 h, suggesting that DNA hydrogel had no cytotoxicity. Also, it was verified in normal breast cells in Supplementary Figure S7B. Then, the non-targeted inhibitory effect of DOX in SK-BR-3 cells was detected, as shown in Supplementary Figure S8; the impact of DOX on cell proliferation was time- and dose-dependent, indicating that DOX therapy is non-targeted and has strong side effects. In control, Figure 2B shows that the viability of SK-BR-3 cells treated with DTA-H/DOX considerably decreased in a time-dependent manner, but no obvious cellular cytotoxicity is detected in MCF-10A cells within 12 h (Supplementary Figure S9).

In addition, DTA-H was found to inhibit the proliferation of SK-BR-3 cells within 48 h (Figure 2B). Because the cell safety of P-RCA alone has been proved in Supplementary Figure S7, we believe that P-RCA alone has no effect on cell proliferation when the treatment time is not more than 48 h. It has been reported that HER2 aptamers can bind to the HER2 on the cell membrane and internalize into cells, induce the degradation of HER2 protein in lysosomes, and indirectly inhibit cell proliferation (Ma et al., 2019), and it is well known that HER2 protein is strongly expressed on the membrane of SKBR-3 cells; thus, we speculate that decoration of HER2 aptamers may play a role in inhibiting cell proliferation.

### Degradation of HER2 Protein Induced by DNA Hydrogel Decorated With Multivalent HER2 Aptamer

The degradation of HER2 protein induced by HER2 aptamer was studied. Firstly, the expression of HER2 protein in SK-BR-3 cells was detected by Western blotting; HER2 protein was strongly expressed in SKBR-3 cells in Supplementary Figure S10. We know that nucleic acid aptamers are easy to degrade, so taking advantage of the high serum stability of DNA hydrogel mentioned above, HER2 aptamers were decorated on DNA hydrogels to enhance their stability and form multivalent HER2 aptamer decorated hydrogels (P-RCA-L-HER2).

**FIGURE 3:**
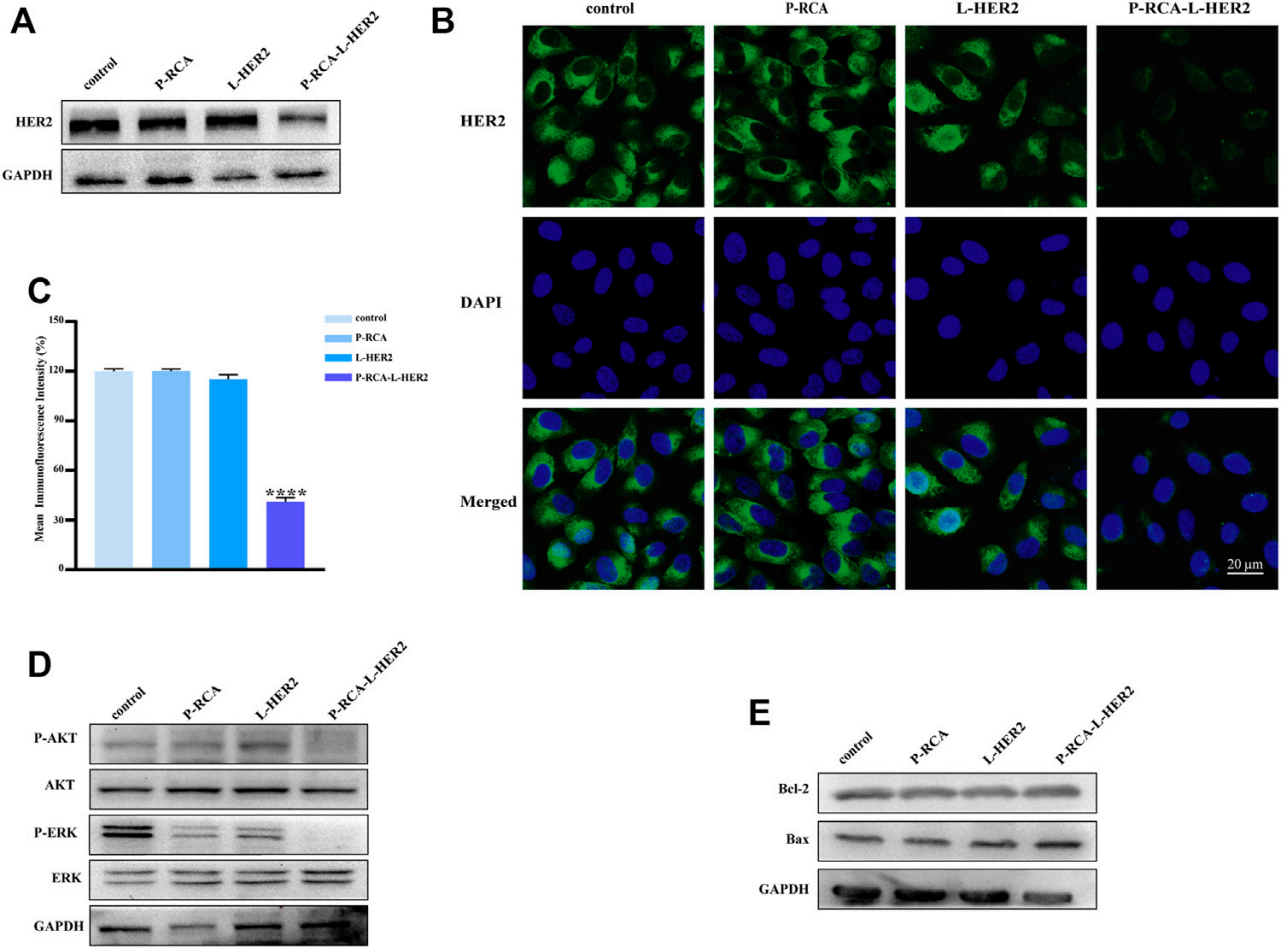
Effects of HER2 aptamer-decorated DNA hydrogel on SK-BR-3 cells. **(A)** Western blotting and **(B)** immunofluorescence of the HER2 protein expression changer of SK-BR-3 cells treated with P-RCA, L-HER2, or P-RCA-L-HER2 for 48 h. Scale bar: 20 μm. **(C)** Quantitative analysis of mean fluorescence intensity of HER2 protein. **(D, E)** Western blotting of the downstream pathway protein of HER2 and apoptosis-related protein expression changes of SK-BR-3 cells treated with P-RCA, L-HER2, or P-RCA-L-HER2 for 48 h. Error bars denote standard deviations from three independent experiments. Statistical analysis: ****p < 0.0001 vs. control.

We compared the effects of single-stranded HER2 aptamers (L-HER2) and P-RCA-L-HER2 on HER2 protein, cell proliferation, and apoptosis by immunoblotting and immunofluorescence. In Figures 3A,B, the expression of the HER2 protein with P-RCA-L-HER2 incubation for 48 h was more significantly inhibited than L-HER2, suggesting that P-RCA-L-HER2 had a stronger binding ability to target cells, thus inducing the significant degradation of HER2 protein. Meanwhile, there was no significant change in the DNA hydrogel incubated group (P-RCA).

It has been reported that the overexpression of HER2 can promote cell proliferation and inhibit apoptosis by activating PI3K/AKT and MAPK/ERK pathways (Yarden and Sliwkowski, 2001). Therefore, the expression of PI3K/AKT and MAPK/ERK pathways and apoptosis-related proteins has been detected. Figure 3D shows that compared with the control, the phosphorylation level of AKT and ERK in the downstream pathway of HER2 decreased after incubation with P-RCA-L-HER2, indicating that the downstream pathway of HER2 was also inhibited. Figure 3E shows that the anti-apoptotic protein Bcl-2 was inhibited, while the expression of pro-apoptotic protein Bax was promoted, indicating that the treatment of multivalent P-RCA-L-HER2 induced apoptosis to some extent. Furthermore, Supplementary Figure S11 shows that dual aptamer-decorated DNA hydrogel (DTA-H) induced more degradation of HER2 protein, indicating that dual aptamer-decorated DNA hydrogel enhanced targeting ability for SK-BR-3 cells. Simultaneously, consistent with the above results, the downstream pathway of HER2 was also inhibited in the DTA-H hydrogel treatment group in Supplementary Figure S11. Surprisingly, we found that Bax significantly increased, and the Bcl-2 and the proliferation-related protein C-myc decreased, indicating that DTA-H could induce apoptosis and suppress proliferation. Therefore, the dual targeting of HER2 aptamer and AS1411 aptamer plays a greatly anti-tumor effect.

### Therapeutic Effect *in vitro* of DTA-H Loaded With DOX

**FIGURE 4:**
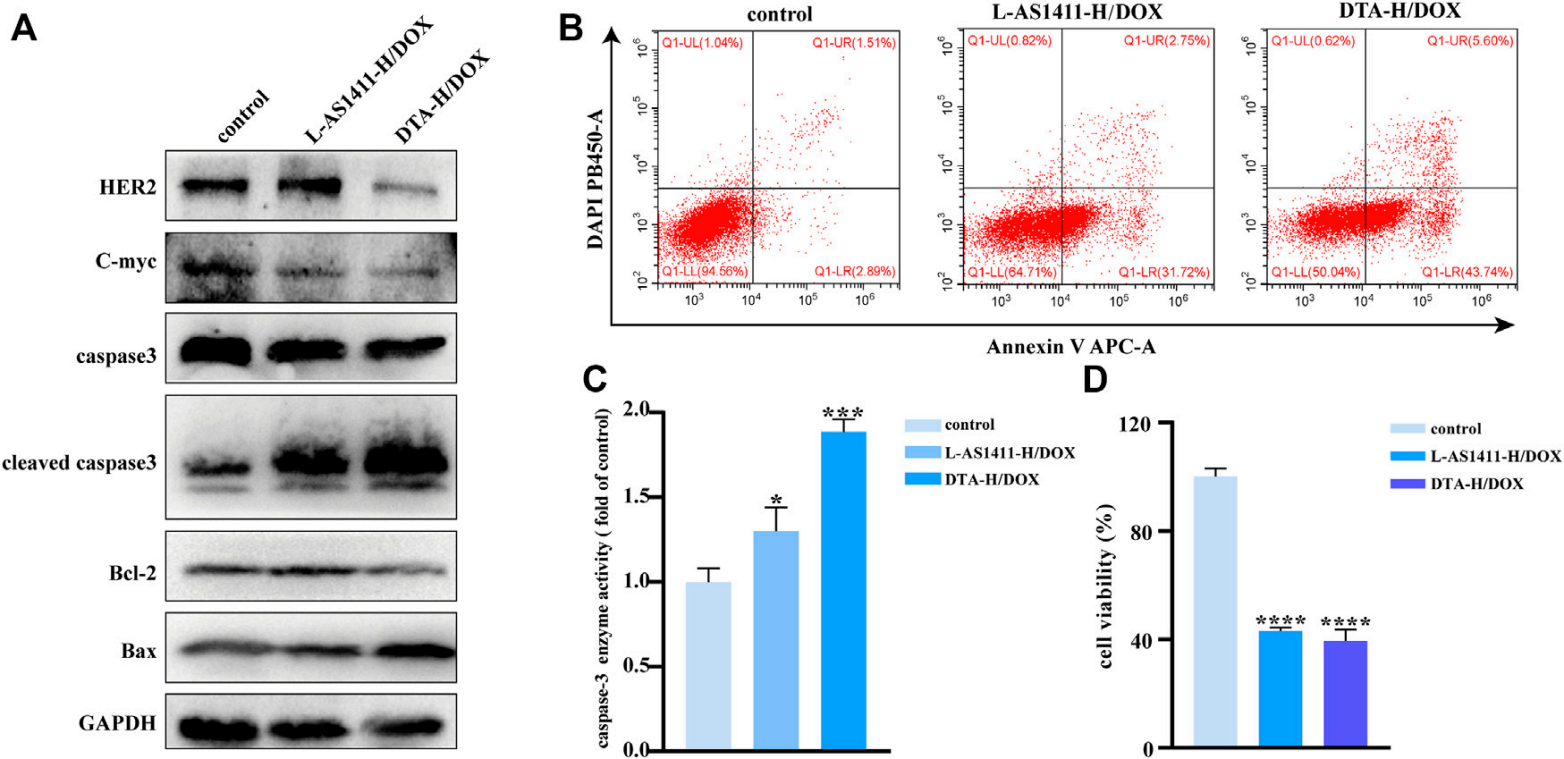
Therapeutic effect *in vitro* of DNA hydrogel loaded with DOX. **(A)** Western blotting of HER2, apoptosis, and proliferation-related protein expression changes of SK-BR-3 cells incubated with L-AS1411-H/DOX or DTA-H/DOX for 24 h. **(B)** Flow cytometry of cell apoptosis of SK-BR-3 cells exposed to L-AS1411-H/DOX or DTA-H/DOX for 24 h. **(C)** Changes in caspase-3 activity and **(D)** cell viability assay (CCK-8 assay) of SK-BR-3 after treatment with L-AS1411-H/DOX or DTA-H/DOX for 24 h. Error bars denote standard deviations from three independent experiments. Statistical analysis: *p < 0.05 vs. control; ***p < 0.001 vs. control; ****p < 0.0001 vs. control.

The therapeutic effects of aptamer-decorated DTA-H loaded with DOX were studied. Figure 4A shows that the expression of apoptotic protein cleavage caspase-3 and Bax was significantly enhanced, while the expression of HER2, Bcl-2, and C-myc were inhibited especially in the DTA-H/DOX group. In addition, after the cells were stained with DAPI and Annexin V, the effect of DTA-H/DOX on apoptosis was studied by flow cytometry. Figure 4B shows that the DTA-H/DOX significantly induced cell apoptosis. However, no effect of DTA-H/DOX on cell proliferation and apoptosis was observed in MCF-10A cells (Supplementary Figure S12). The results of the Calcein staining of Supplementary Figure S13 also showed that cell death increased after the incubation with the DTA-H/DOX for 8 h. The enhanced caspase-3 activity was observed in Figure 4C. Meanwhile, Figure 4D showed that DTA-H/DOX had a stronger inhibitory effect on cell proliferation in SK-BR-3 cells. The above results show that multivalent dual-target aptamer-decorated DNA hydrogel loaded with DOX can significantly induce cancer cell apoptosis or death.

### Anticancer Efficacy *In Vivo* of DNA Hydrogel Loaded With DOX

**FIGURE 5:**
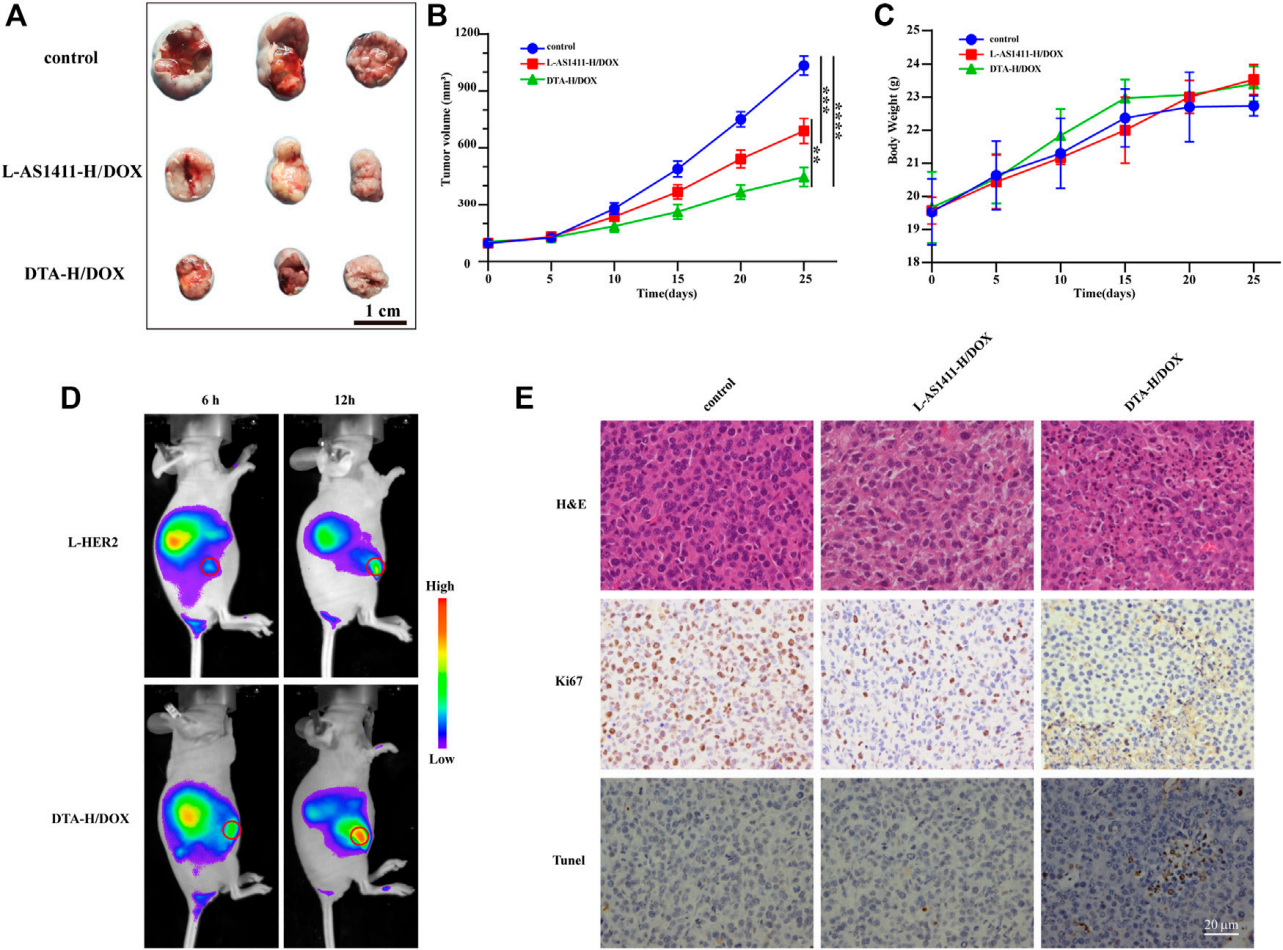
Anticancer efficacy *in vivo* of DTA-H/DOX. **(A)** Tumors peeled from the nude mice at the end of the treatment. Scale bar: 1 cm. **(B)** Tumor growth volume curve during treatment. **(C)** Changes in body weight of mice during treatment. **(D)** Fluorescence imaging *in vivo* after injected by tail vein L-HER2-Cy5 and DTA-H/DOX-Cy5 into mice. The tumors are indicated with red circles. **(E)** H and E, Ki67, and TUNEL staining of the tumor tissues. Scale bar: 20 μm.

Encouraged by the therapeutic effect of DTA-H/DOX *in vitro*, we further evaluated its therapeutic efficacy *in vivo.* Firstly, Cy5-labeled L-HER2 aptamer and DTA-H/DOX were injected by tail vein into SK-BR-3 tumor-bearing mice, and then *in vivo* fluorescence imaging was performed 6 and 12 h later. The fluorescence signal of L-HER2 and DTA-H/DOX can be detected from the liver to the tumor during treatment time, while the fluorescence of the DTA-H/DOX group was stronger than L-HER2 in Figure 5D, providing enhanced specific binding and enough biological stability of DTA-H/DOX. Subsequently, the therapeutic efficacy of DTA-H/DOX *in vivo* was evaluated. The treatment was performed every 4 days, during which tumor size and body weight of mice were measured. In Figures 5A,B, significant tumor growth inhibition was observed in the L-AS1411-H/DOX and DTA-H/DOX groups. Quantitative measurement data by monitoring tumor volume showed that L-AS1411-H/DOX inhibited about 33% of tumor growth, while DTA-H/DOX inhibited about 57% of tumor growth. No noticeable change in body weight was observed in Figure 5C. After the treatment, the tumors of the mice were further collected to evaluate the treatment response. From Figure 5E, hematoxylin and eosin (H and E) staining in the DTA-H/DOX group showed extensive tissue damage. Figure 5E shows that the expression of Ki67 was reduced, and TUNEL staining was enhanced in the DTA-H/DOX group, suggesting that DTA-H/DOX indeed inhibited the proliferation and accelerated the apoptosis of tumors. Furthermore, H and E staining of the major organs (including heart, spleen, liver, lung, and kidney) showed intact cytoplasm and nucleus, and there was no pathological difference compared with the control group (Supplementary Figure S14), confirming the low toxic side effects and the biosafety of DTA-H/DOX. The experimental data demonstrate that DTA-H/DOX possesses enhanced tumor therapeutic efficacy with negligible side effects because of the dual-targeted codelivery of therapeutic nucleic acid and chemodrugs.

## Conclusion

Collectively, we have proposed a multifunctional DNA hydrogel for synergistic therapy of HER2-positive breast cancer with targeted codelivery therapeutic nucleic acid (HER2 aptamer) and chemodrugs (DOX). The proposed DNA hydrogel system has significant advantages in biomedical applications: (1) Dual targeting. The dual targeting of DTA-H was accomplished by two aptamers (HER2 aptamer and AS1411 aptamer) targeting the HER2 and nucleolin protein, which increases the specificity of drug delivery. (2) The multivalence decoration of aptamer. The polyvalent aptamer is decorated on the DNA hydrogel formed by the RCA reaction by simple base complementation pairing. Its advantages are as follows: Firstly, the multivalent aptamer increases the binding affinity between the system and target cells, thus improving the efficiency of drug delivery; secondly, compared with other strategies that amplify aptamers by RCA reactions, our design better preserves the functional secondary structure of aptamers by suspending aptamers on the DNA hydrogel with an additional sequence. In addition, aptamers bound to DTA-H, especially the AS1411, provide rich sites for DOX insertion and improve DOX loading efficiency. (3) Good biostability and biocompatibility. The DTA-H showed stable drug loading capacity to effectively prevent drug leakage and reduce side effects. This synergistic therapy improves the development of HER2-positive or other tumor-targeted nanotherapeutic platforms, which is of great significance in the clinical field.

## Data Availability

The original contributions presented in the study are included in the article/Supplementary Material. Further inquiries can be directed to the corresponding author.
