# Supplementary material for: Dual-Targeted Self-Assembled DNA Hydrogels Decorated With Multivalent Aptamers Loaded With DOX for Anticancer Therapy
Source: Front Pharmacol. 2022 Feb 23;13:807498. doi: 10.3389/fphar.2022.807498 (PMC8905714; doi:10.3389/fphar.2022.807498)
Supplement: Supplementary file 1 [file DataSheet1.docx]

| **name** | **base sequence (5′-3′)** |
| --- | --- |
| **Padlock** | **5’-phosphoraylated-**  **CTGATAAGCTATCCTAGTCGTAACTTGTAGCATCATTCTCCGATTCCGTTCAACATCAGT** |
| **Primer** | **TAGCTTATCAGACTGATGTTGA** |
| **L-HER2** | **GCAGCGGTGTGGGGGCAGCGGTGTGGGGGCAGCGGTGTGGGGTTTTTCTGATAAGCTATCCTAGTCG** |
| **L-AS1411** | **CATCATTCTCCGATTCCGTTTTTTTGGTGGTGGTGGTTGTGGTGGTGGTGG** |
| **HER2 Aptamer** | **GCAGCGGTGTGGGGGCAGCGGTGTGGGGGCAGCGGTGTGGGG** |
| **AS1411 Aptamer** | **GGTGGTGGTGGTTGTGGTGGTGGTGG** |

Supplementary Information

# Supplementary Table

**Table S1.** Sequences of DNA oligonucleotides designed in this work.

# Supplementary Figures

##
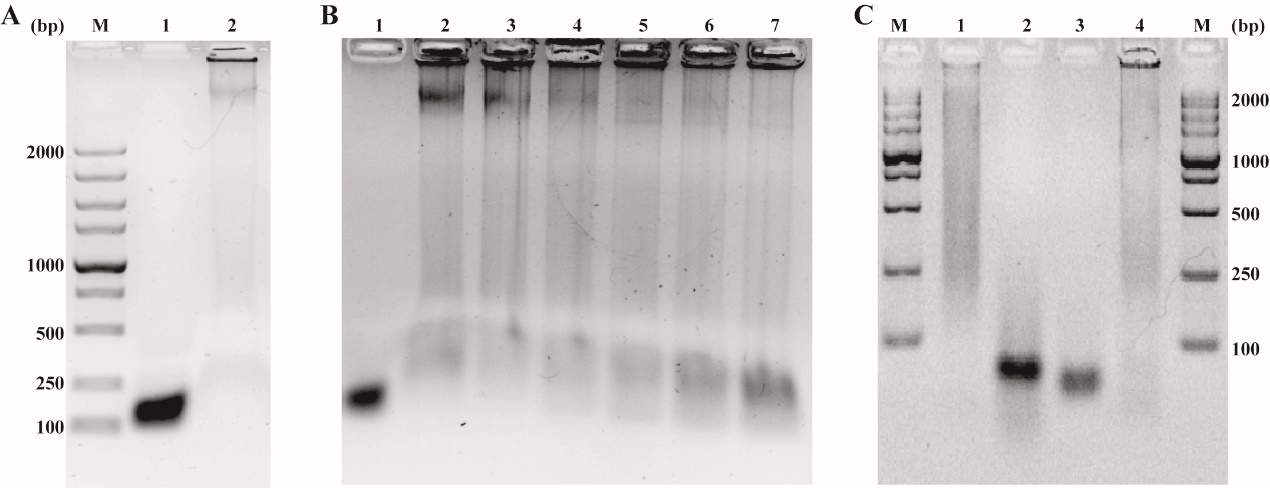


**Figure S1**. Confirmation of the successful synthesis of aptamer-decorated DNA hydrogel. (A) Gel electrophoresis (1%) analysis of RCA product. M represents DNA markers with 2000 bp increments; Lanes 1–2 represent L-HER2 and P-RCA, respectively; (B) Gel electrophoresis (1%) analysis of the assembly of P-RCA and L-HER2. Lanes 1–7 represent L-HER2, P-RCA and the ratio of L-HER2:P-RCA 1:5, 2:5, 3:5, 4:5 and 5:5, respectively; (C) Gel electrophoresis (1%) analysis of the assembly of P-RCA and L-type aptamers. M represents DNA markers with 2000 bp increments; Lanes 1–4 represent P-RCA, L-HER2, L-AS1411 and DTA-H, respectively. The concentrations of P-RCA and L-type aptamers are 10 μM; Proportion is a volume ratio.


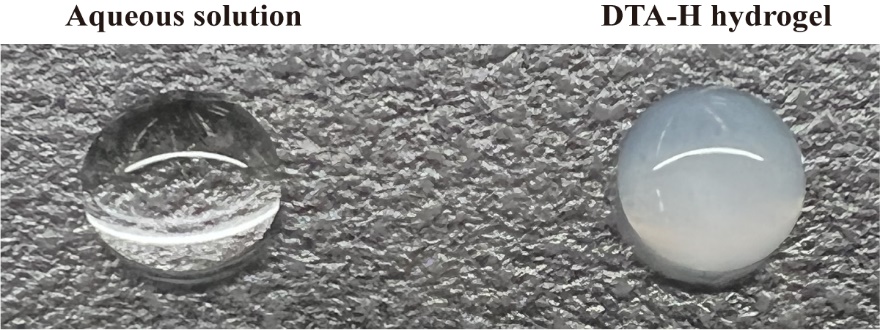


**Figure S2**. The images of DTA-H hydrogel and aqueous solution.

**
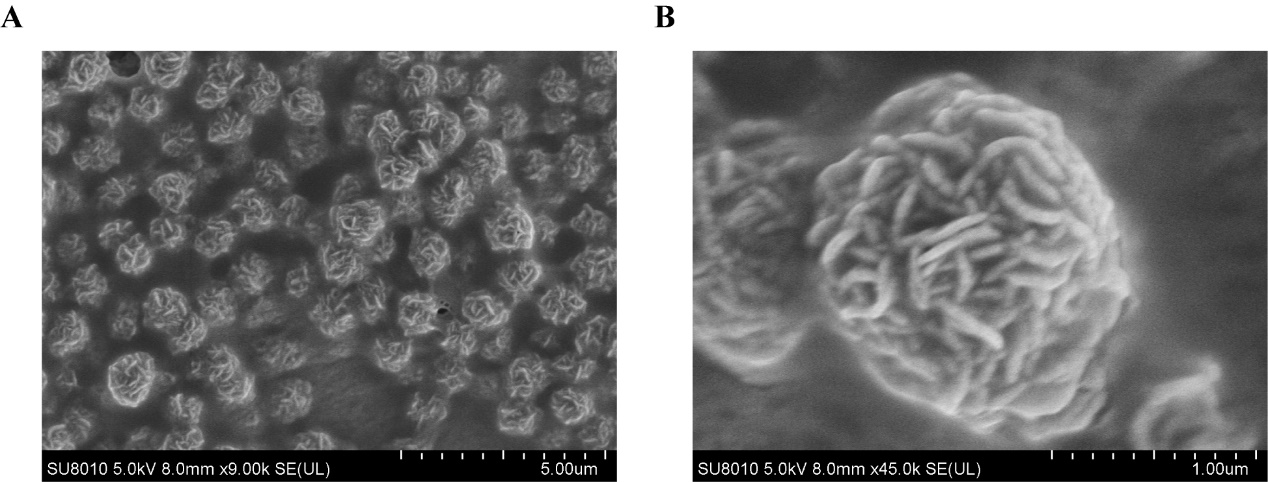
**

**Figure S3**. The SEM images of RCA product. (A) SEM images of P-RCA (scale bar = 5 μm). (B) SEM images of P-RCA (scale bar = 1 μm).

**
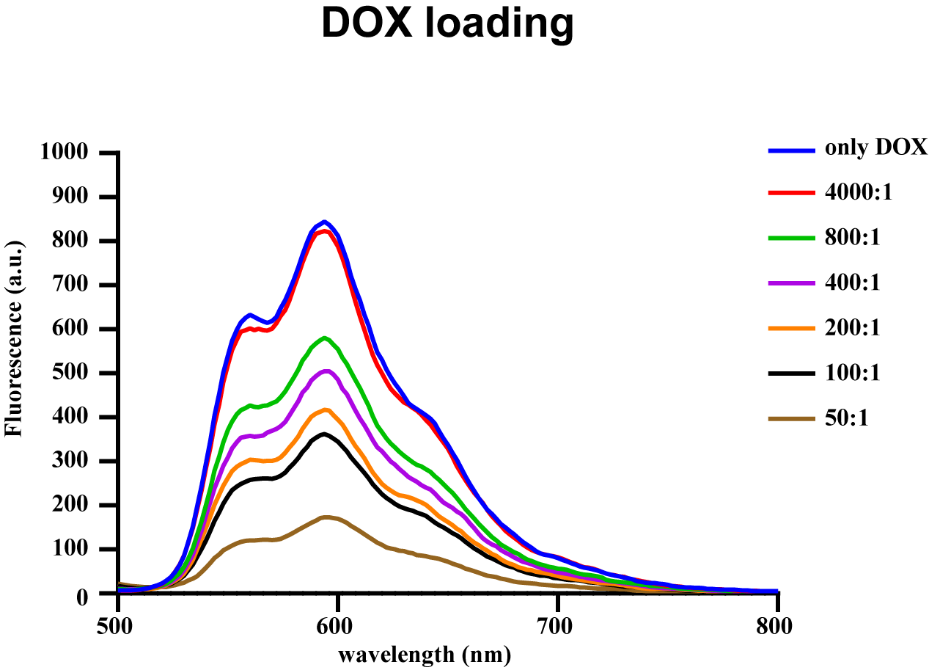
**

**Figure S4**. The fluorescence spectrum of DOX in the presence of increasing amount of DTA-H hydrogel. The concentration of DOX is 50 μM.


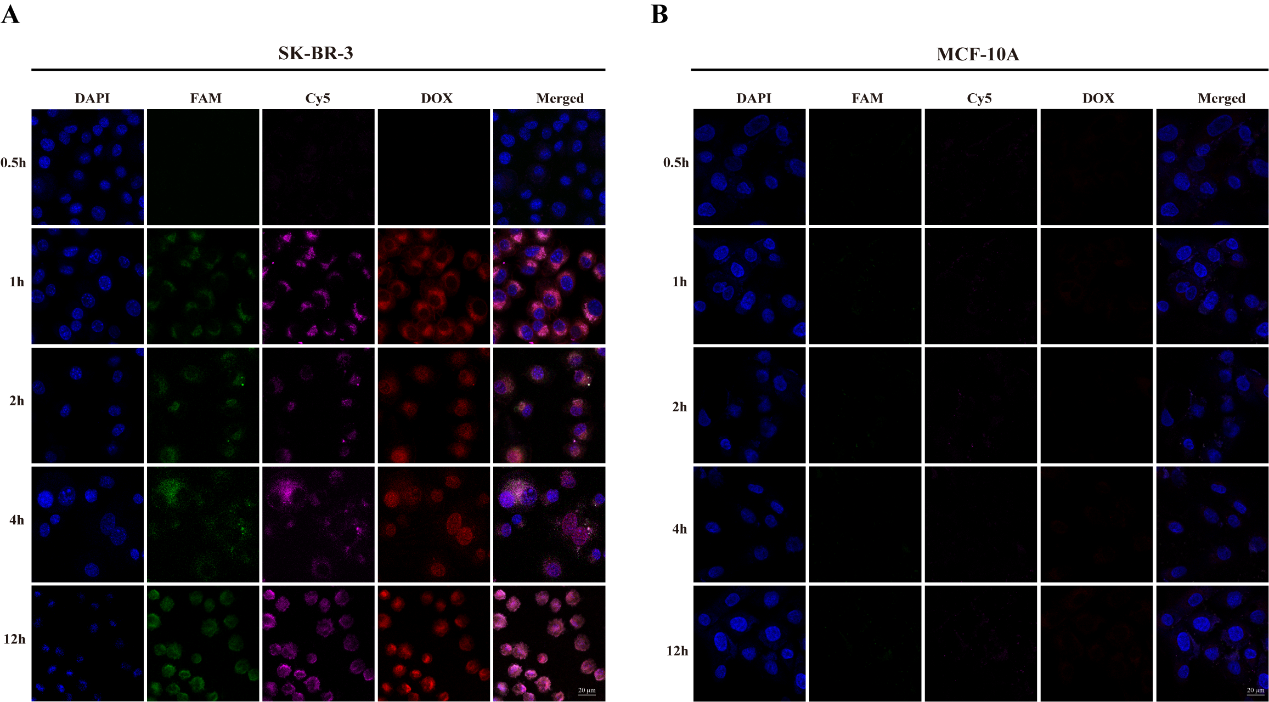


**Figure S5**. Specific internalization of DTA-H/DOX into target cells. Real-time confocal fluorescence imaging of SK-BR-3 cells (A) and MCF-10A cells (B) incubated with DTA-H/DOX for different time. The green fluorescence represents FAM signal (FAM-labeled L-AS1411), purple fluorescence represents Cy5 signal (Cy5-labeled L-HER2) and red fluorescence represents DOX signal, while cell nuclei were stained with DAPI (blue). Scale bar: 20 μm.


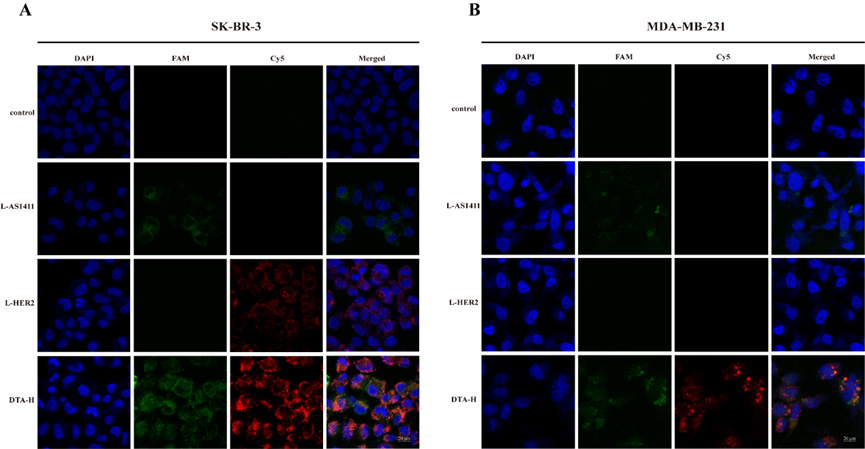


**Figure S6**. Dual targeting of DTA-H to target cells. Confocal fluorescence imaging of SK-BR-3 cells (HER2 and nucleolin positive) and MDA-MB-231 cells (only nucleolin positive) incubated with L-AS1411, L-HER2, DTA-H for 4h. The green fluorescence represents FAM signal (FAM-labeled L-AS1411), red fluorescence represents Cy5 signal (Cy5-labeled L-HER2), while cell nuclei were stained with DAPI (blue). Scale bar: 20 μm.


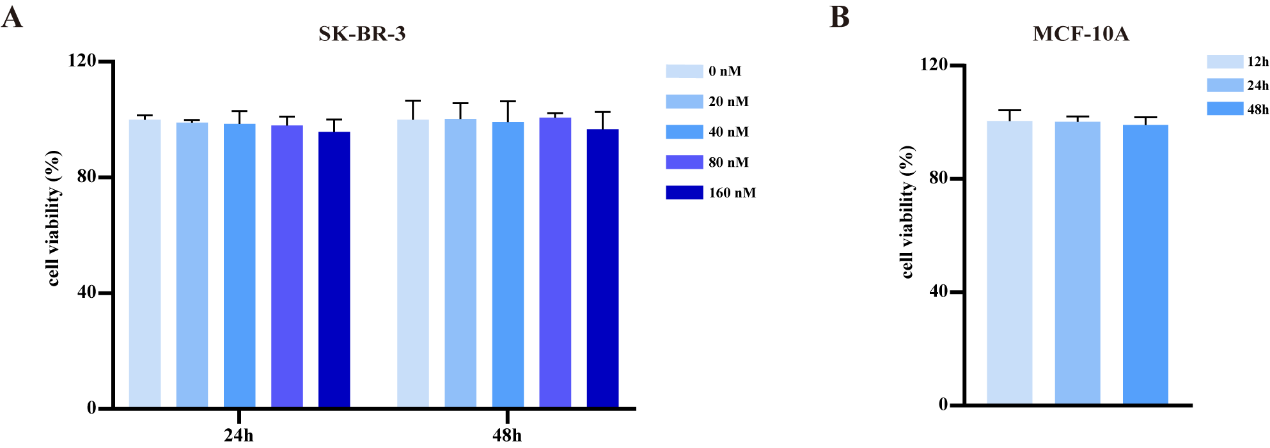


**Figure S7**. Cytotoxicity of P-RCA to SK-BR-3 cells (A) and MCF-10A cells (B). (B)The incubation concentration of P-RCA was 160 nM. Error bars denote standard deviations from three independent experiments. Statistical analysis: * p < 0.05 vs control; *** p < 0.001 vs Control.


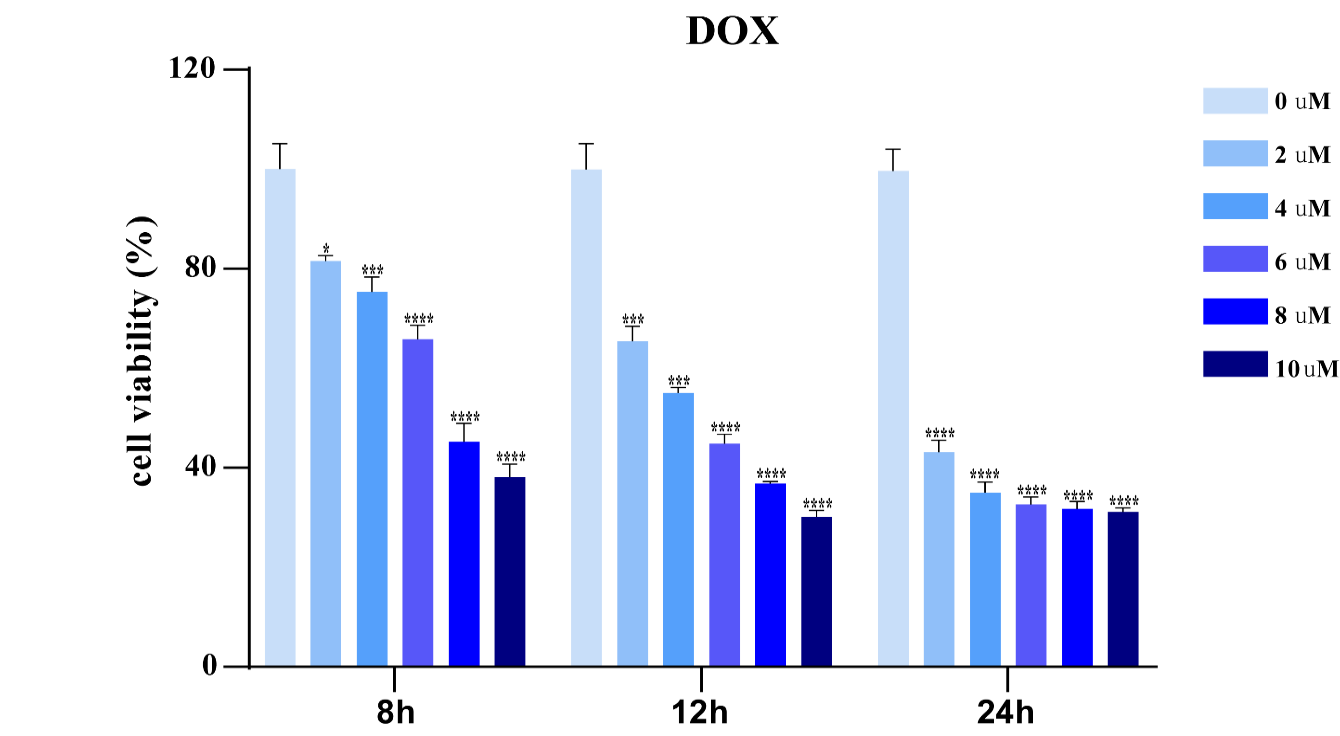


**Figure S8**. Cytotoxicity of DOX to SK-BR-3 cells. Error bars denote standard deviations from three independent experiments. Statistical analysis: * p < 0.05 vs control; *** p < 0.001 vs Control; **** p < 0.0001 vs Control.


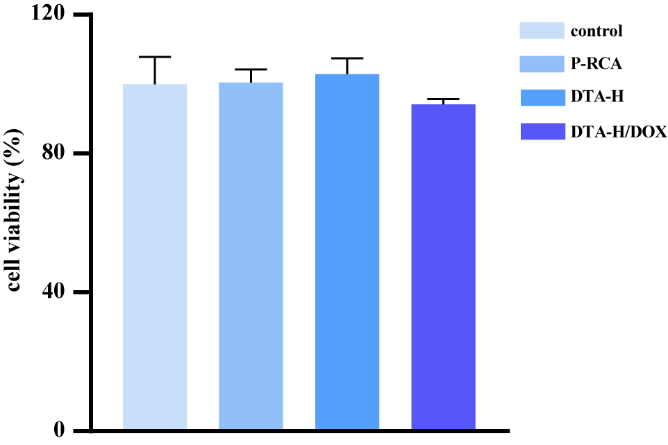


**Figure S9**. Cytotoxicity of P-RCA, DTA-H and DTA-H/DOX to MCF-10A cells. The incubation time was 12 h. Error bars denote standard deviations from three independent experiments. Statistical analysis: * p < 0.05 vs control; *** p < 0.001 vs Control.


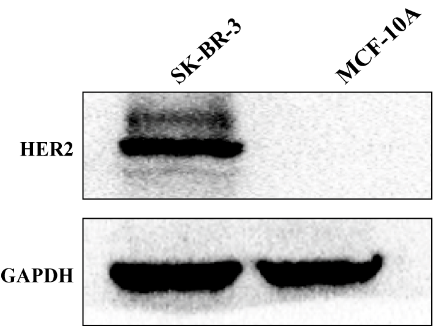


**Figure S10**. The expression of HER2 protein in SK-BR-3 and MCF-10A cells.


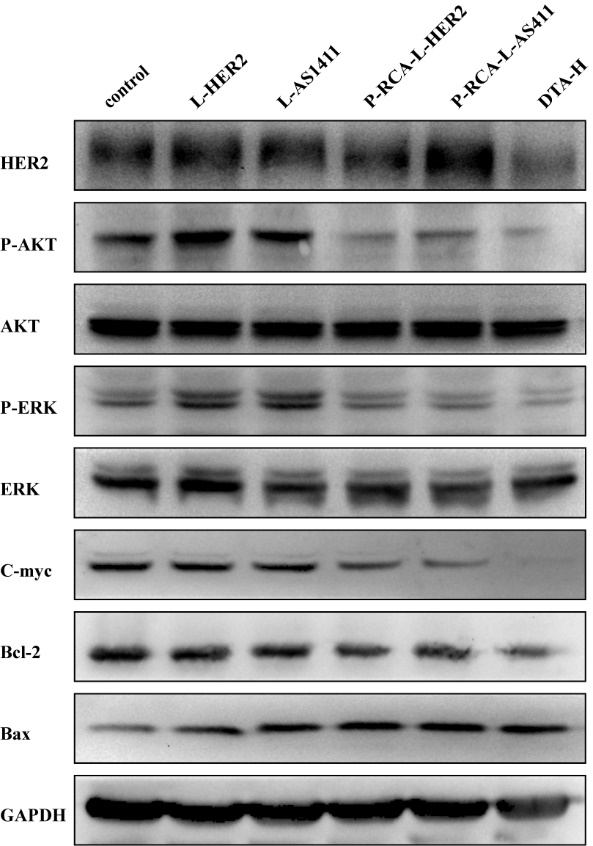


**Figure S11**. Effects of DTA-H on SK-BR-3 cells. Western blotting analysis of HER2, the down pathway of HER2, apoptosis or proliferation related protein expression level after SK-BR-3 cells were treated with P-RCA, L-HER2, L-AS1411, P-RCA-L-HER2, P-RCA-L-AS1411 or DTA-H for 48 h.


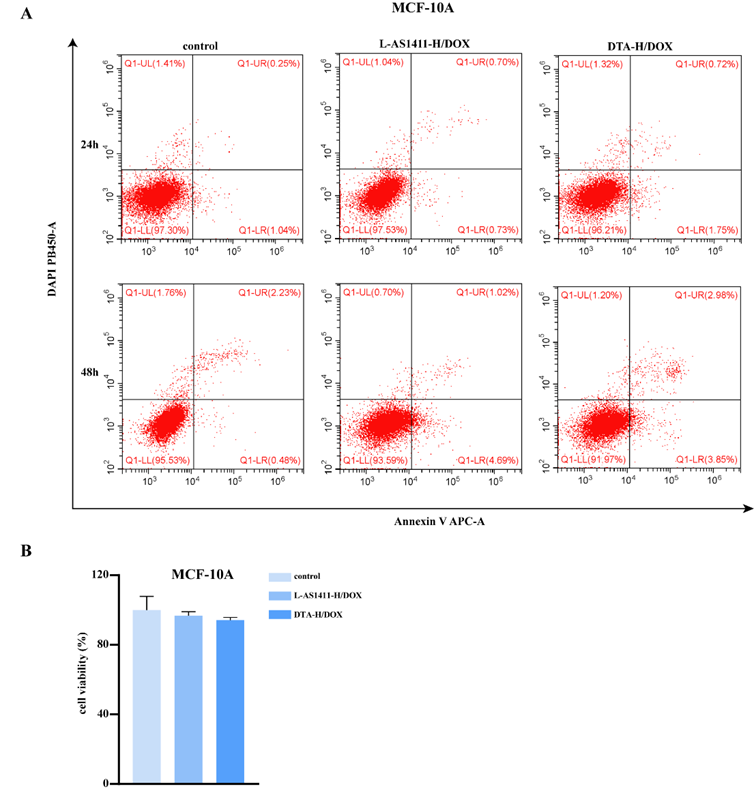


**Figure S12**. Cytotoxicity of L-AS1411-H/DOX and DTA-H/DOX to MCF-10A cells. (A) Flow cytometry analysis of cell apoptosis after MCF-10A cells exposed to L-AS1411-H/DOX or DTA-H/DOX for 24 h and 48h. (B) Cell viability assay (CCK-8 assay) analysis of cell proliferation after MCF-10A cells exposed to L-AS1411-H/DOX or DTA-H/DOX for 12 h. Error bars denote standard deviations from three independent experiments. Statistical analysis: * p < 0.05 vs control; *** p < 0.001 vs Control.


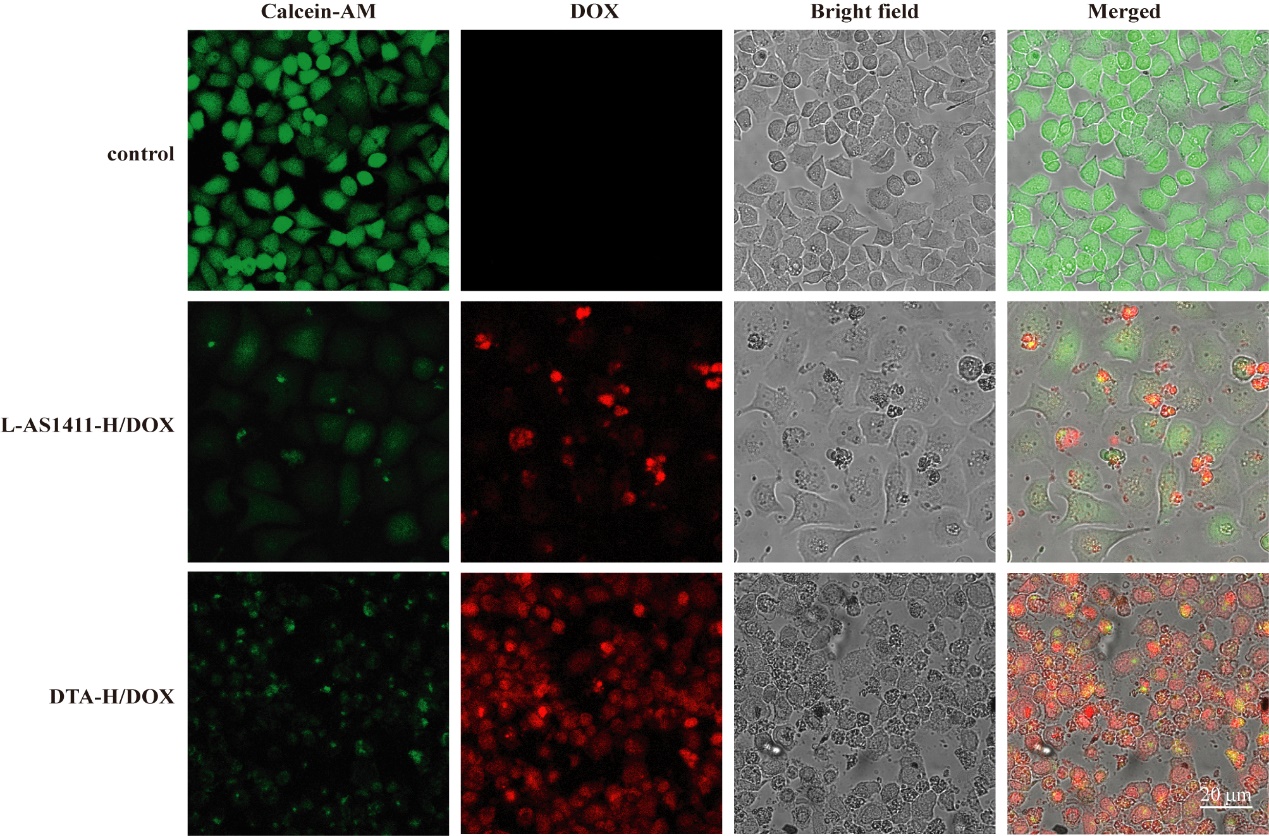


**Figure S13**. The confocal fluorescence imaging of Calcein-AM staining on SK-BR-3 cells after treated with L-AS1411-H/DOX and DTA-H/DOX for 8h. The concentration of DOX is 4 μM. The red fluorescence points represent the DOX entered cells, while the green fluorescence points represent the live cells. Scale bar: 20 μm.


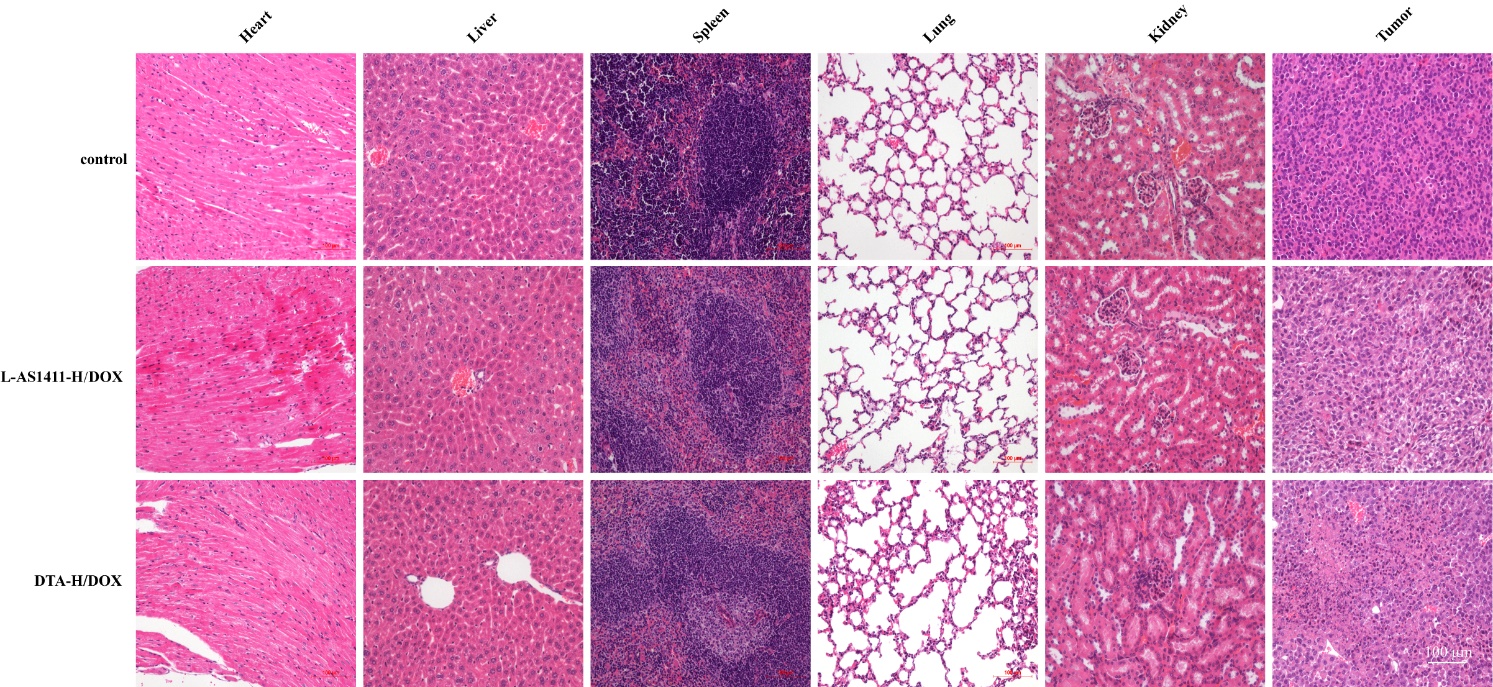


**Figure S14**. Images of H&E staining from the major organs (heart, liver, spleen, lung, and kidney) and the tumors of mice treated with L-AS1411-H/DOX and DTA-H/DOX. Scale bar: 100 μm.
